# Mesquite Gum as a Novel Reducing and Stabilizing Agent for Modified Tollens Synthesis of Highly Concentrated Ag Nanoparticles

**DOI:** 10.3390/ma9100817

**Published:** 2016-10-04

**Authors:** Maira Berenice Moreno-Trejo, Margarita Sánchez-Domínguez

**Affiliations:** Centro de Investigación en Materiales Avanzados, S. C. (CIMAV), Unidad Monterrey, Alianza Norte 202, Parque de Investigación e Innovación Tecnológica, 66628 Apodaca, Nuevo León, Mexico; maira.moreno@cimav.edu.mx

**Keywords:** silver nanoparticles, NP synthesis, mesquite gum, modified Tollens method

## Abstract

The synthesis that is described in this study is for the preparation of silver nanoparticles of sizes ranging from 10 nm to 30 nm with a defined shape (globular), confirmed by UV-vis, SEM, STEM and DLS analysis. This simple and favorable one-step modified Tollens reaction does not require any special equipment or other stabilizing or reducing agent except for a solution of purified mesquite gum, and it produces aqueous colloidal dispersions of silver nanoparticles with a stability that exceeds three months, a relatively narrow size distribution, a low tendency to aggregate and a yield of at least 95% for all cases. Reaction times are between 15 min and 60 min to obtain silver nanoparticles in concentrations ranging from 0.1 g to 3 g of Ag per 100 g of reaction mixture. The proposed synthetic method presents a high potential for scale-up, since its production capacity is rather high and the methodology is simple.

## 1. Introduction

Metallic nanoparticles usually show unique and improved properties (which are size- and shape-dependent) compared to its version in bulk due to their high surface-to-volume ratio [1,2]. In the particular case of silver nanoparticles (NPs), the intensive research arises from the fact that they have multiple applications such as catalysis [3,4], spectrally selective coatings for solar energy absorption [5,6], medical imaging [7,8] and antimicrobial sterilization [9]. This has caused the need to create synthesis methods for this kind of nanomaterial in order to satisfy the nanotechnology market. Thus, several methods have been designed [1,2,4,8,10,11], but most of them (conventional chemical and physical methods) are expensive and/or they require the use of aggressive chemicals and/or specialized equipment.

Currently, the use of “green” methods is more desirable because some reactants used in chemical methods are hazardous to the environment [10,11]; in the specific case of silver nanoparticles, the “green” aspect of the method consists in the appropriate selection of a solvent media, reducing agent, and non-toxic stabilizing agent that are friendly with the environment as described in the work of Abou El-Nour and Tai [2,12]. Some of these agents can be replaced by molecules produced by microorganisms such as bacteria, fungi, yeasts, algae or plants [13] to make them more ecofriendly. Extracts of some plants (leaf, flower, seed, tuber, bark and sap) have been extensively explored and compared to other process that were inspired in other kind of biological organisms [9,14,15,16,17,18,19,20,21,22]. When “green” synthesis techniques and Tollens reaction are combined, it can result in the formation of silver nanoparticles with controlled size as seen in the literature [23,24,25,26]. In the modified Tollens reaction, the Ag^+^ ions are reduced by sugars or polysaccharides in the presence of ammonia, typically producing stable silver nanoparticles with a size range from 50 to 200 nm as shown by Saito et al. [24], in this particular case glucose was employed as the reducing agent and the concentrations of AgNO_3_ were set between 0.012 and 0.047 mol/L. In general, the concentration of AgNO_3_ used in modified Tollens studies was rather low (0.001 to 0.01 mol/L). In some related investigations, the presence of surfactants was required in order to stabilize these particles, as is in the work of Kvítek and Soukupova [27,28], in which two surfactants were used, namely sodium dodecyl sulfate (SDS) and polyoxyethylene (20) sorbitan monooleate (Tween 80), as well as polyvinylpyrrolidone (PVP 360); in the work of Yu et al. [29], silver nanoparticles of various morphologies were synthesized by adjusting the concentrations of n-hexadecyl trimethyl ammonium bromide (HTAB) and Tollens reagent [Ag(NH_3_)_2_]^+^, at 120 °C in a modified silver mirror reaction. Different modifications to Tollens reaction can produce various kinds of silver particles as reported by Kvitek et al. [26]; they produce silver hydrosols with a size from 20 to 50 nm and different shapes depending on the reducing agent (d-glucose, d-fructose, d-xylose, d-maltose and methyl adenine) that is employed. Similarly, the group of Panáček et al. [30] developed silver nanoparticles with controllable sizes by reduction of [Ag(NH_3_)_2_]^+^ with two monosaccharides (glucose and galactose) and two disaccharides (maltose and lactose) in combination, obtaining particles with a size range from 25 to 450 nm under the modified Tollens method.

In the particular case of our study, the reducing and stabilizing agent originates from the mesquite tree (*Prosopis velutina*), which is a plant that naturally can produce a proteinaceous arabinogalactan gum that is similar to Arabic gum. There is no report on the synthesis of nanoparticles using mesquite gum. This tree species is abundantly present in the desert parts of Mexico and the south of USA. In Mexico, the sap of the three is collected as dried pearls and packed for selling to the public as a candy. Some chemical research has determined that this gum is a mixture of polysaccharides and glycoproteins that are cross-linked forming arabinogalactan proteins. This kind of proteins give some physicochemical properties (immunochemical identity and high solubility in water) to the gum to form compact globular structures; these structures in aqueous solution provide the capacity to reduce the surface tension and to act as a steric stabilizer [31,32,33,34].

In the present work, we present the results of a synthesis technique (modified Tollens process) for the preparation of silver nanoparticles using mesquite gum (*Prosopis velutina*) as a steric stabilizer and reducing agent in aqueous silver nitrate solutions as an improvement of the traditional method. Through this method silver NPs size can be controlled, exhibiting a narrow size distribution according to DLS characterization, stability through time, and a good potential for scaling-up, since very high precursor (AgNO_3_) concentrations can be used, compared to other works (up to 50 to 500 times more concentrated). The natural mesquite gum used for this synthetic route was subjected to a purification process and characterized, which is also described in this work.

## 2. Results and Discussion

Since some decades ago, the Tollens process has been used for the deposition of metallic silver on some objects to make them look like mirrors, but recent research has also proven that it works as a method for producing silver nanoparticles with a controlled size [23,24,25,26,27,30,35]. The Tollens reaction implies the reduction of [Ag(NH_3_)_2_]^+^_(aq)_, the Tollens reagent, by an aldehyde (-RCHO) or a carbohydrate (commonly glucose). The reaction is described as follows:

(1) $${{\left[Ag{\left(NH_{3}\right)}_{2}\right]}^{+}}_{\left(aq\right)}+RCHO_{\left(aq\right)}\to Ag_{\left(s\right)}+RCOOH_{\left(aq\right)}$$

Commonly, when the silver complex ion solution is mixed with the reducing agent solution in a clean glass vial, a silver mirror film forms on the inside within a few minutes at certain temperature. In the case of the modified Tollens process of this study, when the reducing agent and stabilizer solution (mesquite gum) is added to the silver complex ion solution, stable dispersions of silver nanoparticles, rather than mirror films, could be obtained. In the correct temperature conditions, the solution went from clear and colorless at the time of mixing to a yellow-brownish color. The final products were very dark yellow or brown silver colloid dispersions with narrow size distribution and an excellent colloidal stability. The transition from transparent solutions to brown dispersions was fast, taking 1 to 3 min. Mason et al. reported a similar color change for their studies on the bioreduction mechanism of silver ions into nanoparticles by switchgrass extract in a 10^−3^ M AgNO_3_ solution [11]. The reaction of their system takes more than twice the time reported in the present study, and very diverse shapes of silver nanoparticles with a size range between 20 and 40 nm were obtained, with a tendency to agglomerate.

The characteristics of the silver nanoparticles obtained by the present method (size, size distribution and stability) depend on the concentration of precursor, the temperature and time of the reaction, and the ratio of AgNO_3_: mesquite gum during the reduction process. In contrast, other studies require additionally the presence of surfactants in the solution of silver nanoparticles in order to achieve stability and a narrow size distribution [23,27,28].

### 2.1. UV-Vis Absorption Spectroscopy

**Figure 1:**
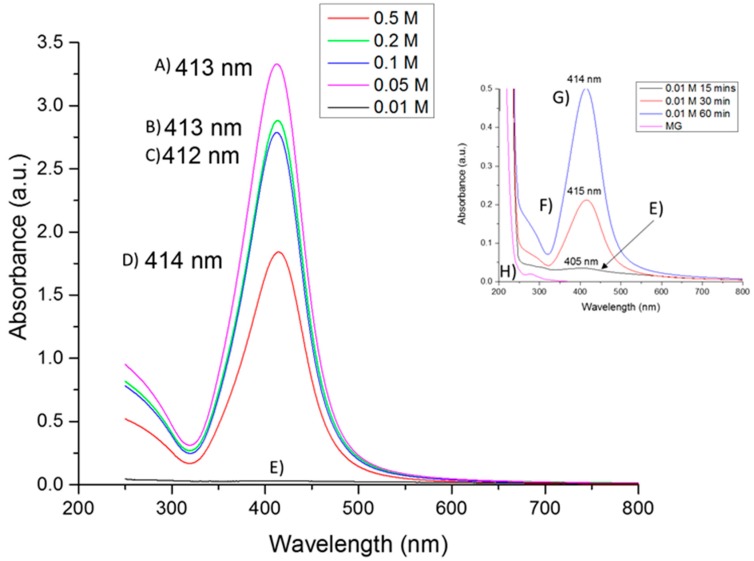
UV-VIS absorption spectra of silver nanoparticles with different initial AgNO_3_ concentrations: (**A**) 0.5 M diluted 1:1000 15 min treatment; (**B**) 0.2 M diluted 1:1000 15 min treatment; (**C**) 0.1 M diluted 1:1000 15 min treatment; (**D**) 0.05 M diluted 1:100 15 min treatment; (**E**) 0.01 M diluted 1:10 15 min heat treatment; (**F**) 0.01 M diluted 1:10 30 min heat treatment; (**G**) 0.01 M diluted 1:10 60 min heat treatment; and (**H**) Mesquite Gum, same conditions as 0.01 M diluted 1:10, 15 min heat treatment sample. The mentioned dilutions were only for measurement of UV-Vis spectra, after Ag NPs synthesis.

UV-visible spectroscopy is one of the most used techniques for optical characterization of silver nanoparticles. The optical absorption spectra of metal nanoparticles are characterized by surface plasmon resonance (SPR), which moves towards longer wavelengths with increasing particle size [36]. The UV-vis absorption spectrum of the colloidal solution of silver nanoparticles (Figure 1) shows a typical optical absorption band of a surface plasmon with a maximum around 413 nm for each of the samples synthesized using AgNO_3_ concentrations of 0.01, 0.05, 0.1, 0.2 and 0.5 mol/L (15 min, 1:1 Precursor:Gum). Similar bandwidth suggests similar polydispersity in terms of particle size distribution. The localization of the surface plasmon band at around 413 nm in all cases may be attributed to non-covalent interactions of the stabilization conferred by mesquite gum [37]. In addition, SPR bands (wavelength, position and FWHM) depend on size, size distribution, shape, lattice parameters and filling factors of the metal particles [38]; thus, it can be assumed that the samples share several of these characteristics, arising from the use of the same wet-chemical synthesis method [39]. The presence of a unique peak indicates the presence of spherical or globular silver nanoparticles, because if the number of peaks increases it means that the symmetry of the nanoparticle decreases [40]. Characterization by SEM and STEM confirmed this assumption. In addition, the presence of the minimum at ~320 nm in all samples corresponds to the wavelength at which the real and imaginary parts of the dielectric function of silver almost fade [41].

The absorbance of a sample is directly proportional to the concentration of the absorbing species in the solution [42]. In the specific case of Figure 1E, the concentration was rather low (0.01 M diluted 1:10, also the heat treatment was only 15 min). In addition, several components of the mesquite gum possess absorption bands in the 200–700 nm region, such as glucose [43], glucuronic acid [44] and galactose [45]. This can be observed in the MG (mesquite gum) spectrum (Figure 1H), this sample was run for comparison under the same conditions of sample (E) (same composition and treatment, but without Ag precursor). The same increase in absorption intensity at the short wavelength zone of the spectrum was observed in Figure 1H. Comparing the sample shown in Figure 1E to the samples synthesized using also 0.01 M AgNO_3_ but using higher reaction time (30 or 60 min at 80 °C, Figure 1F,G, respectively) we can conclude that the reaction run only 15 min at 80 °C (Figure 1E) was an incomplete reaction, because the samples shown in Figure 1F,G exhibit a behavior more consistent with the samples carried out at higher AgNO_3_ concentration (Figure 1A–D). Thus, for the sample with insufficient reaction time, the bands arising from mesquite gum become more important, and the absorption at shorter wavelengths becomes higher than the plasmon band. Increase of absorption intensity also indicates that the amount of silver nanoparticles increases (as seen on the inset with samples (Figure 1F,G)). Furthermore, as mentioned above, the plasmon band and the full width at half maximum (FWHM) depends on the extent of colloid aggregation [46,47,48]. The stable position of absorbance peak indicates that particles do not aggregate, or at least that they aggregate to the same extent [48]. This could be due to the use of the same ratio of mesquite gum: AgNO_3_ in all cases. Under the conditions of the samples (Figure 1A–G) the aggregation of silver nanoparticles was not highly developed; this is consistent with the fact that the hydrodynamic diameter by DLS remained stable as a function of time. In the case of the sample shown in Figure 1E, some colloidal aggregation may have been promoted, as shown by the size dependence as a function of time. Finally, it is concluded that the strong surface plasmon resonance centered at ~413 nm clearly indicates the formation of well dispersed Ag NPs, such as in other works [49,50].

### 2.2. Morphological Analysis: SEM and STEM

**Figure 2:**
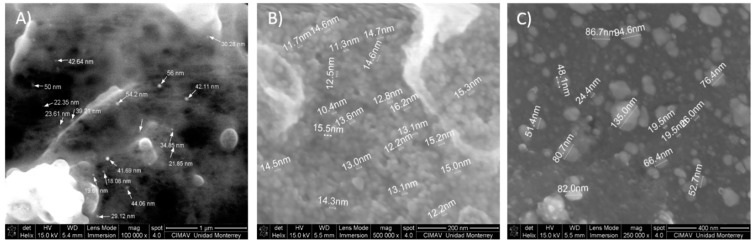
Micrographs of silver nanoparticles synthesized at different AgNO_3_ concentrations: (**A**) concentration 0.01 mol/L (15 min, 1:1 AgNO_3_:MG); (**B**) concentration 0.2 mol/L (15 min, 1:1 AgNO_3_:MG); and (**C**) concentration 0.5 mol/L (15 min, 1:1 AgNO_3_:MG).

Figure 2 shows typical SEM micrographs of silver nanoparticles deposited onto a carbon tape, however the low electric conductivity of the stabilizing agent (mesquite gum) lowers the resolution of the image at higher magnification. Figure 2A shows 0.01 mol/L (15 min, 1:1 AgNO_3_:MG) sample, it was observed that for this sample the silver nanoparticles were embedded in a layer of mesquite gum; in addition, the interaction of the equipment with the sample causes some lifting of the organic material. These artifacts are caused by the heat produced during the measurement, because mesquite gum is an organic molecule. The size range in Figure 2A goes from 18 nm to 56 nm and the morphology that is appreciated can be described as spherical. Since for this sample (0.01 mol/L, 15 min, 1:1 AgNO_3_:MG), Ag nanoparticles were embedded inside the mesquite gum, the particle size results obtained by SEM are not reliable, since it is likely that smaller, non-agglomerated particles embedded in the mesquite gum cannot be observed under these conditions.

As the concentration increases, like in the case of the samples synthesized with AgNO_3_ 0.2 mol/L (15 min, 1:1 AgNO_3_:MG) (Figure 2B) and 0.5 mol/L (15 min, 1:1 AgNO_3_:MG) (Figure 2C), the nanoparticles get closer to each other tending to form agglomerates; but the formation of these agglomerates is likely to have occurred during isolation of the solid nanoparticles for SEM analysis, since, as shown in Section 2.3 (DLS results), the smallest particle size population was dominant, and only a small percentage of particles volume was present as large agglomerates. The sample with 0.2 mol/L AgNO_3_ concentration (15 min, 1:1 AgNO_3_:MG) presented particle sizes ranging from 4 to 20 nm, and the sample with 0.5 mol/L (15 min, 1:1 AgNO_3_:MG) AgNO_3_ has a size range from 20 nm to 80 nm, plus agglomerates of over 100 nm. As seen in Puchalski et al. and Mason et al. [11,51], even commercial silver nanoparticles or those synthesized with plant extracts tend to agglomerate. When the agglomerates where examined, it was observed that those large particles consisted of smaller particles (3–15 nm). Again, agglomeration must have occurred during isolation of the solid samples for SEM analysis as DLS analysis of these samples showed relatively low polydispersity index.

**Figure 3:**
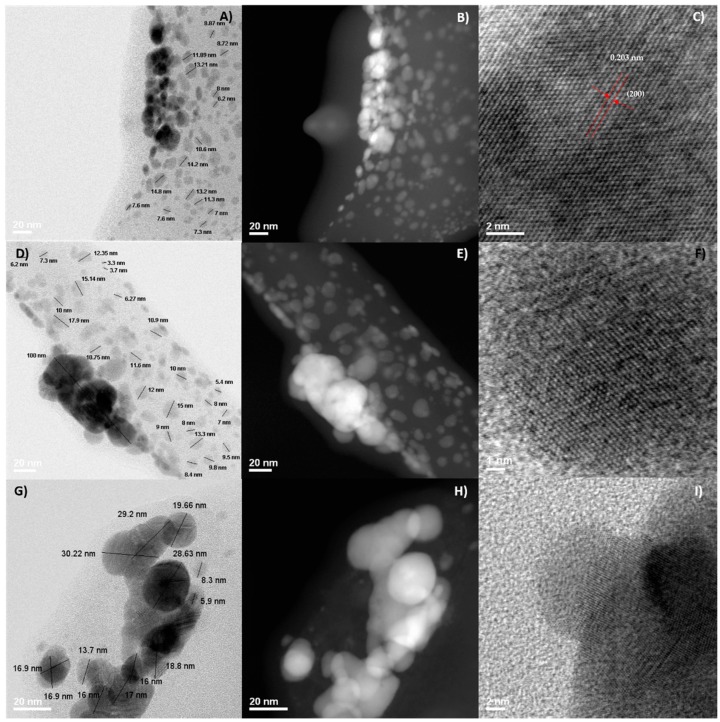
STEM micrographs of silver nanoparticles concentration 0.01 M, 15 min, 1:1 AgNO_3_:MG: (**A**) bright field; (**B**) dark field; and (**C**) measurement of interplanar distance for a particular nanoparticle. Silver nanoparticles concentration 0.2 M, 15 min, 1:1 AgNO_3_:MG: (**D**) bright field; (**E**) dark field; and (**F**) crystalline features. Silver nanoparticles concentration 0.5 M; 15 min, 1:1 AgNO_3_:MG: (**G**) bright field; (**H**) dark field; and (**I**) crystalline features.

In agreement with the SEM micrographs, STEM analysis (Figure 3) confirmed that the size and morphology of the resulting silver nanoparticles depend of the AgNO_3_ initial concentration (Figure 3A–I). In addition, the synthetized nanoparticles tend to agglomerate in the formvar-carbon coated copper grid (agglomeration occurred during isolation for SEM and STEM characterization) and they were coated by a mesquite gum layer as shown better in the dark field image in Figure 3B,E,H. Particles with a size range from 3 nm to 15 nm were obtained, in coexistence with agglomerates ranging from 20 nm to 80 nm, for the sample synthesized with a concentration of 0.01 mol/L AgNO_3_ (Figure 3A–C). We measured the interplanar distances in some micrographs of representative nanoparticles; as an example, Figure 3C shows a nanocrystal in which the interplanar distance is 0.203 nm, associated to plane (220) corresponding to the fcc structure of silver. Samples synthesized with AgNO_3_ 0.2 M (Figure 3D–F) and 0.5 M (Figure 3G–I) presented larger particle sizes and agglomerates, as observed in the micrographs. Crystalline features were also confirmed for these samples (Figure 3F,I).

### 2.3. Colloidal Stability Assesment: DLS

In order to analyze the colloidal stability of silver nanoparticles that are dispersed in the aqueous media, the effective hydrodynamic diameter of the particles was measured via DLS and the results are listed in Table 1 for the samples at different conditions; results are shown in volume percent. As expected, the effective hydrodynamic diameter increases as the concentration of the sample is higher. Size data are reported in Table 1. Particle size obtained by SEM and STEM are in the same range as the particle size of the main population obtained by DLS; an average size from SEM and STEM was not attempted due to uncertainties arising from the presence of mesquite gum which did not allow very high quality images. The polydispersity index was relatively low, in the order of 0.2, except for some of the samples, which presented values up to 0.3.

The presence of a second peak observed in some of the samples indicates the appearance of large aggregates (4 μm or higher) and are attributed to possible agglomerates of mesquite gum that are not completely dissolved or agglomerates of silver particles, or a mixture of these. In any case, this second peak corresponding to agglomerates represents the less abundant population. For lower concentrations (0.01 M AgNO_3_), the heat treatment time was increased because the precursor concentration was so small that the reaction kinetics were too slow. It was found that this slightly increases the average particle size. In addition, reducing the ratio of AgNO_3_: mesquite gum (from 1:1 to 1:0.3) causes an increase in the polydispersity index, generating the presence of larger agglomerates and increasing the standard error of the analysis.

After one month of synthesis, a series of measurements were performed in order to follow the stability of the samples. The results of these measurements are shown in Table 2. At first instance, we can observe the beginning of the destabilization for the solutions of lower concentration (0.01–15 min present an increase of 2.7% of volume in the second peak, and 0.05–15 min presents also an increase in the second peak, in this case of 3.9%). This could be attributed to either Ostwald ripening, an effect of degradation of the gum or agglomerates of gum or nanoparticles, because these samples contained lower concentration of gum (as lower AgNO_3_ concentration uses lower gum concentration, whilst keeping the AgNO_3_:MG ratio constant at 1.1). In contrast, the other samples (with higher concentration of both gum and AgNO_3_) are stable and consistent with its corresponding first DLS measurement.

Similarly, in a lapse of three months, the samples showed similar sizes as in the firsts measurements (except for the samples with the smaller AgNO_3_ concentration (0.01 mol/L)). The average effective hydrodynamic diameter after three months is approximately 30 nm remaining stable from the first measurement with a narrow distribution; this is valid for the concentrations greater than 0.01 mol/L, which have an average size of 10 nm coexisting with a small volume of larger agglomerates. Lack of sediments or growth of aggregates indicates that for AgNO_3_ concentrations above 0.01 mol/L, the silver NPs dispersion achieved an effective steric stability conferred by the presence of mesquite gum that becomes attached or linked to the surface of the silver nanoparticles. It is actually quite remarkable that the sample with the highest AgNO_3_ concentration (0.5 mol/L) presented a very stable hydrodynamic size (around 32 nm), a PDI of only about 0.2, and only one particle size population after one and three months of synthesis. Most likely, the population of agglomerates present right after synthesis may be attributed to agglomerated mesquite gum which after some equilibration time was properly dissolved in the reaction media. This appeared to be a general trend, since at the beginning (Table 1) seven samples presented a small population of agglomerates, whereas after three months (Table 3) only three samples presented such population of agglomerates.

### 2.4. Infrared Analysis

**Figure 4:**
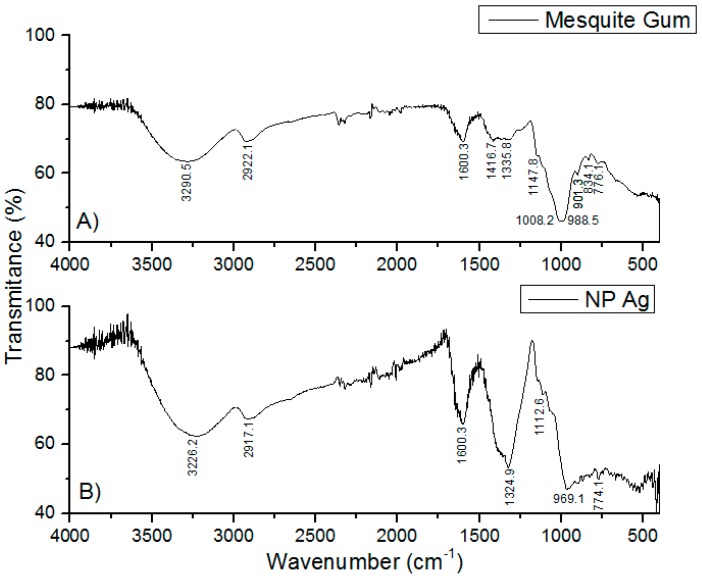
IR spectra of: (**A**) solid powder of mesquite gum; and (**B**) dried 0.5 mol/L 15 min 1:1 silver NPs.

The FTIR spectra of the solid MG showed absorptions of O–H and C–H at 3290 and 2922 cm^−1^ (Figure 4A), respectively. A band centered near 1600 cm^−1^ is assigned to amide I attributed to the protein content of the samples [52]. COO^−^ asymmetric stretching bands located at 1416 cm^−1^ were also identified. The bands around 1150 cm^−1^ and 900 cm^−1^ can be attributed to vibration modes of C–O and the C–O–H groups of carbohydrates (such as glucose, mannose and galactose), according to the literature [31,52,53,54]. The bands at 834 and 776 cm^−1^ indicate the occurrence of pyranose glycosidic acetal groups [54]. A shift in the absorbance peaks was observed (Figure 4B) from 3290 to 3226 cm^−1^ and from 2922 to 2917 cm^−1^, suggesting the binding of silver with hydroxyl and carboxylate groups. Furthermore, the occurrence of the peak at 1324 cm^−1^ and disappearance of the peaks at 1416, 1008, and 834 cm^−1^ confirm that the reduction of the silver ions is coupled to the oxidation of the hydroxyl and carbonyl groups, indicative of a more extensively oxidized nature of the gum [55]. Based on the band shift in the hydroxyl and carbonyl groups and the loss of existing carbonyls and occurrence of a new carbonyl peak, it can be inferred that both hydroxyl and carbonyl groups of the gum are involved in the synthesis of silver nanoparticles. Such variations of the FTIR spectra in the shape and peak position of the hydroxyl and carboxylate groups have been reported, where silver nanoparticles were synthesized using another polysaccharide, gum Acacia (Arabic gum) [55,56]. Supplementary Materials (Figures S1–S3), corresponding to characterization data of Mesquite gum and Arabic gum showed that both gums have similar structure and behavior.

### 2.5. Silver Ion Analysis (ISE Measure)

Aliquots of the Ag NPs colloids were measured with a silver ion-selective electrode in order to investigate if complete reduction was achieved, by determining residual Ag^+^ ions in the colloidal solution, the results are shown in Table 4; an ISA (Ionic Strength Adjustor) was employed. As can be seen in all cases, AgNO_3_ was reduced at ~99% for the formation of Ag nanoparticles proving the high production capacity of the process described in this investigation. The relative amount of Ag**^0^** contained in the samples is determined by subtracting the ionic silver concentration from the total silver concentration. The total silver concentration is calculated from the experimental data and the ionic silver concentration is measured using the ISE.

(2) $$\%Ag^{0}=\left(\left(Total\;silver\;\left(M\right)-Ionic\;silver\;\left(M\right)\right)\times 100\right)/Total\;silver\;\left(M\right)$$

### 2.6. Discussion

**Figure 5:**
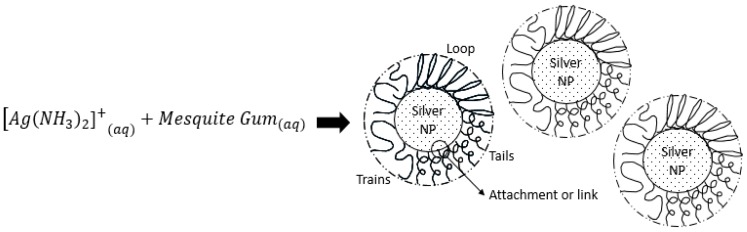
Schematic diagram of steric stability conferred to silver nanoparticles by the mesquite gum.

The reaction conditions including heating time, AgNO_3_ concentration, and AgNO_3_: Mesquite gum ratio had a certain influence on the size, polydispersity, shape and stability of silver nanoparticles. In this work, we have demonstrated that, by using mesquite gum, the aqueous diamine silver complex could be reduced by a modified Tollens reaction method, as explained further on in this section, generating silver nanoparticles. Based on UV-Vis, SEM, STEM and DLS analysis, and previous references of the potential of mesquite gum for use in general synthesis [31,57] a mechanism of silver nanoparticles formation and growth under the previously explained experimental conditions is proposed. Adsorption of the mesquite gum on the surface of silver nanoparticles plays a key role in the stabilization and controlled growth of the nanoparticles by providing a steric stability, as shown in the schematic diagram of Figure 5.

**Figure 6:**
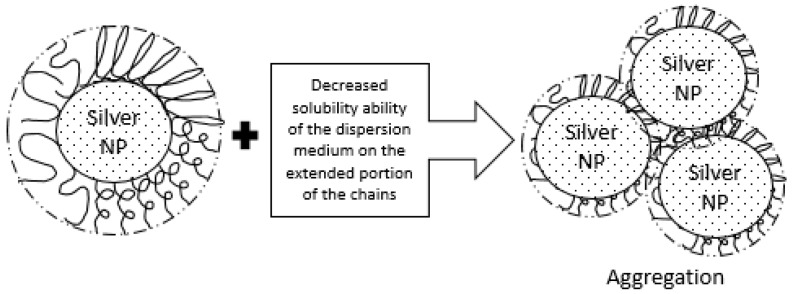
Schematic diagram of purposed mechanism of aggregation.

In the Tollens reaction, [Ag(NH_3_)_2_]^+^ ions accept electrons from the mesquite gum molecule producing Ag**^0^** atoms which form seeds for the further formation of Ag nanoparticles. The large chains of the mesquite gum as a natural polysaccharide (containing tannins, galactose, arabinose, rhamnose, glycoproteins, amino acids, and glucuronic acid) prevent excess growth, adsorbing themselves to the surface of silver nanoparticles, which results in small dimensions and a narrow size distribution of the Ag NPs. This stable layer of mesquite gum around the silver nanoparticles prevents them from aggregation and works as stabilizer. If there is some aggregation, such as in the case of the sample with a concentration of 0.01 mol/L initial AgNO_3_, it may be caused by the decreased ability of the dispersion medium to solubilize the extended portions of the mesquite gum chains, which may cross the point of flocculation, as explained in the schematic diagram shown in Figure 6. Alternatively, the low concentration of mesquite gum in this sample may induce desorption of gum after some time, leading to destabilization. In addition, degradation of gum would cause more flocculation problems in such diluted samples.

**Figure 7:**
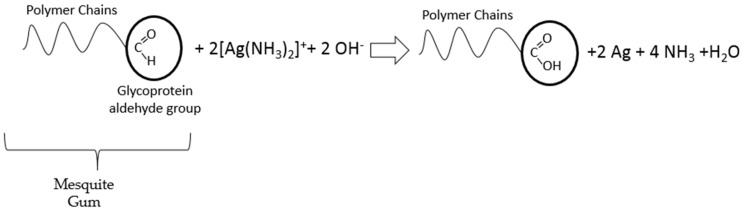
Schematic diagram of proposed reduction reaction.

As previously mentioned, the polymeric chains of the mesquite gum stabilize the nanoparticles, but they also have a key role in the reduction process. Mesquite gum has a composition which is similar to Arabic gum [33,58] (comparisons and evaluations to support this statement are in Supplementary Materials, Figures S1–S3 and Tables S1 and S2); thus, it is constituted by high-molecular-weight glycoproteins (~90% carbohydrates) and low-molecular-weight heterogeneous polysaccharides that contain many hydroxyl and carbonyl groups in the polymer chains [56]. Thus, the reduction process may be attributed to the hydroxyl and/or the aldehyde groups of the mesquite gum. In the case of hydroxyl groups, this deduction is based on the literature [56,59,60] which mention that the hydroxyl groups of poly(ethylene glycol) (PEG) or ethylene glycols can act as reducing agents [59]. These hydroxyl groups of the mesquite gum reduce the silver ions in the diamine silver complex (presenting a behavior similar to PEG) to form silver nanoparticles through an oxidation mechanism as reported elsewhere [61]. On the other hand, aldehyde groups from the aldehyde lactone part of the glucuronic acid [62] may act in a similar manner to the classic Tollens reaction with the aldehyde group from glucose, as shown in Figure 7. This is supported by FTIR analysis (Section 2.4), with the corresponding variations in the FTIR spectra (shape and peak position of the hydroxyl and carbonyl groups).

### 2.7. Comparison with Other Methods (Production Capacity)

Most of the methods reported in the literature for the synthesis of silver nanoparticles use rather low concentrations of silver precursor (typically AgNO_3_). The main reason for this is that Ag nanoparticles synthesized in aqueous media tend to agglomerate, even when stabilizers are used; additionally, low precursor concentration is used in order to obtain a small particle size. Thus, typical AgNO_3_ concentrations used are in the millimolar range, which leads to production capacity in the order of 0.01–0.1 g of Ag nanoparticles per 100 g of reaction mixture. Thus, several research groups have attempted to improve this production capacity.

Table 5 summarizes data for two types of studies for comparison with the present investigation: some approaches use gums or a Tollens method as reported here, while other approaches are rather different, but they are included in the table because of their good production capacity. Note that although reaction time is reported in Table 5, the “production capacity” has been estimated taking into account only the grams of Ag nanoparticles that can be obtained in 100 g of reaction mixture.

Firstly, it must be pointed out that by using 0.5 mol/L solution of AgNO_3_ in the present method, the estimated production capacity (for Ag nanoparticles in the order of 30 nm) is approximately 3 g of Ag nanoparticles per 100 g of reaction mixture; in addition, the process takes less than 30 min and no special equipment is required. The work of He et al. is an example of a modified Tollens approach, which as in the classical “silver mirror” method, uses glucose as reducing agent [25]. The modification is that Al(NO_3_)_3_ is added, which under the reaction conditions forms Al(OH)_3_, and this induces the formation of plate-like silver nanoparticles. The estimated production capacity in that study is about 0.43 g of Ag nanoparticles per 100 g of reaction mixture, which is almost an order of magnitude smaller than the present study.

The works by Kora et al. and Dong et al. [55,63,64] use gums that have certain similarities to mesquite gum; in such cases, instead of using the Tollens approach, the gum is just mixed with AgNO_3_ in water and temperature is increased (in the works by Kora et al., an autoclave is used for heating). The precursor concentration used is rather low, so the production capacity is in the order of 0.01–0.06 g of Ag nanoparticles per 100 g of reaction mixture; furthermore, the process take about 0.5–1 h by using the autoclave or 3 h with normal heating at 60–80 °C. The advantage of such works is that only the gum, AgNO_3_ and water are needed, which could be desirable for certain applications.

The study by Sosa et al. uses a W/O microemulsion as confined reaction media [65]. Although the production capacity is relatively high (1.5 g of Ag nanoparticles per 100 g of reaction mixture), this method uses an aromatic solvent (toluene) and a relatively high surfactant concentration (about 30%). Nonetheless, the particle size obtained in that work is smaller (below 10 nm), so this method may be a good choice when a smaller size and a high production capacity is needed. In the work by Melendrez et al., the production capacity is also relatively high at about 1 g of Ag nanowires per 100 g of reaction mixture, however the solvent used is ethylene glycol (whereas the present method is aqueous); in addition, a microwave treatment is needed, which hinders its perspectives for scale-up [66].

In the investigation by He et al., a production capacity of 0.54 g of Ag nanoparticles per 100 g of reaction mixture was estimated, however in this approach a synthesized gemini surfactant is used, which is not commercially available for industrial production; thus, perspectives for scaling-up are low [67]. The work by Li et al. can achieve up to 3.2 g of Ag nanoparticles per 100 g of reaction mixture [68], which is similar to our study, however, high surfactant concentration (95%) and a high reaction time (1–3 days) at 100 °C is needed, which in terms of materials and resources (time and energy consumption) is less competitive than the method proposed here.

Finally, in the work by Toisawa et al., a very high production capacity is achieved, in the order of 2–9 g of Ag nanoparticles per 100 g of reaction mixture [69]. This method uses Ag_2_O powder as precursor, ultrasound, and it is carried out during several hours (3–10 h) in a mixture of ethanol and toluene. Thus, although the production capacity is very high, the use of solvents and several hours of ultrasound also may hinder scaling-up perspectives of this method.

As mentioned in Section 2.5, the production capacity of the present strategy was confirmed by performing a silver ion analysis in all samples; the low values of Ag^+^ obtained correspond to high values of Ag**^0^**, and hence a very efficient reduction reaction by the modified Tollens process proposed here was achieved.

## 3. Experimental Section

### 3.1. Materials and Methods

Silver nitrate (99%, Sigma Aldrich), sodium hydroxide (Sigma Aldrich), ammonium hydroxide (28%–30% (*w*/*w*), Sigma Aldrich) were used for the preparation of silver NPs without any further purification. Mesquite gum samples from *Prosopis velutina* were collected manually in the form of exuded pearls in the Mexican state of Sonora by local suppliers, and a batch was purchased in a local convenient store (Mieles de Sonora, Hermosillo, Mexico) and purified in the laboratory.

### 3.2. Mesquite Gum Purification

The mesquite exuded pearls were selected and cleaned following the method described in previous works [31,57,70], only the best pearls were selected (class MGA and MGB), followed by pulverization in a mortar, after that the powder was dissolved in distilled water at room temperature for 24 h; once that time passed for hydration of the gum, the liquid was filtered with a Whatman no. 2 filter paper and then the filtered solution was frozen for 15 h and lyophilized in a FreeZone (Labconco) for 26 h.

### 3.3. Synthesis of Silver Nanoparticles

The experiment was performed for various silver nitrate concentrations (0.01, 0.05, 0.1, 0.2 and 0.5 mol/L). Then the solution of silver nitrate was precipitated with an appropriate amount of a 10% solution of sodium hydroxide. The brownish precipitate of Ag_2_O was dissolved with a solution of ammonium hydroxide that was added dropwise with continuous stirring until a transparent solution of silver ammonium complex was formed. Then, a certain amount of dissolved mesquite gum was added to the mixture at room temperature with gentle stirring. Finally, the reactions were kept under continuous stirring for different time intervals (15, 30 and 60 min) at 80 °C. As a result, a brown-yellow solution was formed, indicating the formation of silver nanoparticles.

### 3.4. Characterization

The morphology of the nanoparticles was investigated by scanning electron microscopy (SEM) Nova Nano 200 FEI (FEI Company, Eindhoven, The Netherlands), the samples for SEM analysis were precipitated with isopropanol, filtered onto 0.22 µm Millipore filters and washed with deionized water. The silver NPs were mounted onto an aluminum stub using conductive carbon tape and examined at 20 kV. The UV-visible absorption spectra of the silver nanoparticles colloidal dispersions were measured using a spectrophotometer Cary 5000 UV-Vis-NIR (Varian Inc., Palo Alto, CA, USA) at wavelengths 200–800 nm; appropriate dilutions were prepared for such measurements. STEM images were collected on a 200 keV JEOL JEM2200 FS electron microscope (JEOL Ltd., Tokyo, Japan), the samples for STEM characterization were prepared by placing a drop of a colloidal silver dispersion on a formvar-carbon coated copper grid which was dried at room temperature, then they were washed with several drops of deionized water in order to remove mesquite gum as much as possible. The diameter and size distributions of silver nanoparticles in solution were assessed by dynamic light scattering (DLS, Malvern Zetasiezer Nano-ZS, Malvern Instruments, Worcestershire, UK), also stability as a function of time (up to 3 months) was measured. Infrared spectroscopy was performed in order to identify organic functional groups and to find the molecular interactions and bonds between the silver nanoparticles and mesquite gum. The powders of mesquite gum and silver NPs were directly run in a Thermo Nicolet 6700 FT-IR spectrophotometer and measurements were carried out. In addition, a silver/sulfide ion specific electrode (ISE) (Silver/Sulfide ionplus Sure-Flow Solid State Combination Ion Selective Electrode, Cat. No. 9616 BNWP, Thermo Scientific, Waltham, MA, USA) was used in combination with a pH/mV meter (pH/ISE meter Orion Star™ A214, Thermo Fisher Scientific, Rockford, IL, USA) to measure free Ag^+^ ions of the samples; an AgNO_3_ calibration was performed first. A linear response and a *R*^2^ of 0.995 was achieved.

## 4. Conclusions

We have successfully synthesized silver nanoparticles employing purified mesquite gum (*Prosopis velutina*), which is produced by a tree that is highly abundant in the desert zone of México and the southern United States. Mesquite gum acts as a reducing and stabilizing agent in a modified one-pot Tollens reaction. The presence of mesquite gum promotes the formation of stable silver nanoparticles. Using this method, the silver nanoparticles can be easily prepared and they can be employed in many applications, because this rapid and facile synthesis method permits the control of particle size, long shelf life (good colloidal stability through time), and a narrow size distribution according to DLS analysis.

The results show that Ag particles are in the nanometric scale with a globular shape, and this is confirmed by SEM and STEM images, where depending of the initial concentration of AgNO_3_ and some other conditions such as AgNO_3_: Mesquite gum ratio and reaction time, it is possible to obtain particles with the average size of 10 nm or 30 nm; further studies with AgNO_3_ concentrations between 0.01 and 0.05 M may result in intermediate values of size range. Following the effective hydrodynamic diameter with DLS analysis as a function of time, it can be concluded that these silver nanoparticles have a good colloidal stability and exhibit a low tendency to aggregate. This feature can be used to efficiently decorate other materials such as catalysts supports.

The most important aspect of the method reported here, is its unusually high production capacity, together with its simplicity and the green aspect of raw materials used. With the initial concentration of 0.5 mol/L AgNO_3_, which is the maximum reported here, it can yield up to 3 g of Ag nanoparticles per 100 g of reaction mixture. As shown in Table 5, most methods report production capacities much lower than this value; in the cases where production capacity is comparable, excess solvents, surfactants, reaction time and/or energy consumption (use of microwave or ultrasound) is needed. In addition, the method reported here is aqueous, the reducing and stabilizing agent is a natural compound which only needs a very simple purification to be used, the reaction times are short, the temperature used is relatively low and only needed for a few minutes, and the equipment used is very simple. Ammonia and sodium hydroxide left in the reaction media can be easily eliminated by coagulating the particles with isopropanol one time, followed by redispersion in water, to obtain basically Ag nanoparticles stabilized by mesquite gum and dispersed in water, with a good colloidal stability. Thanks to these characteristics and the resulting stability of the Ag nanoparticle dispersions obtained, a great potential for scaling-up is envisaged.

## Figures and Tables

**Table 1:** Measurements of the effective hydrodynamic diameter (DLS data) of Ag NPs samples synthesized at different conditions.

Sample Silver Concentration (mol/L)	Time in Treatment (min)	AgNO_3_:Mesquite Gum Ratio	PdI	SIZE (d.nm) ± SE (% Volume)	
				1st Peak	2nd Peak
0.01	15	1:1	0.159	10.3 ± 3.1 (99.4)	65.9 ± 20.6 (0.6)
0.05	15	1:1	0.225	27.9 ± 11.3 (95.9)	4674 ± 1009 (4.1)
0.1	15	1:1	0.252	35.4 ± 13.9 (86.2)	4585 ± 1053 (13.8)
0.2	15	1:1	0.205	32.8 ± 13.5 (100)	-
0.5	15	1:1	0.221	35.9 ± 13.9 (94)	4920 ± 889.7 (6)
0.01	30	1:1	0.232	15.9 ± 3.1 (95.2)	67.4 ± 18.1 (4.8)
0.01	60	1:1	0.175	12.3 ± 2.5 (93.8)	60.4 ± 21.4 (6.2)
0.05	30	1:1	0.169	33.2 ± 7.5 (100)	-
0.05	60	1:1	0.315	26.9 ± 4.1 (100)	-
0.2	15	1:0.3	0.276	28.9 ± 12.9 (96.1)	4965 ± 866.3 (3.9)
0.2	15	1:0.5	0.306	22.9 ± 14.6 (100)	

**Table 2:** Follow-up of the colloidal stability of Ag NPs samples synthesized at different conditions after one month of synthesis: hydrodynamic diameter (DLS data).

Sample Silver (mol/L)	Time in Treatment (min)	AgNO_3_:Mesquite Gum Ratio	PdI	Size (d.nm) ± SE (% Volume)	
				1st Peak	2nd Peak
0.01	15	1:1	0.739	9.9 ± 7.6 (96.5)	4329 ± 1172 (3.3)
0.05	15	1:1	0.225	27.7 ± 10.1 (92)	4946 ± 881.1 (8)
0.1	15	1:1	0.252	30.4 ± 12.1 (100)	-
0.2	15	1:1	0.205	31.6 ± 11.2 (100)	-
0.5	15	1:1	0.221	31.4 ± 10.7 (100)	-
0.01	30	1:1	0.396	11.4 ± 3.4 (91)	56.06 ± 37.5(5.6)
0.01	60	1:1	0.391	10.3 ± 1.5 (98.1)	5283 ± 711.4 (1.9)
0.05	30	1:1	0.194	31.0 ± 11.5 (100)	-
0.05	60	1:1	0.315	28.0 ± 9.9 (99)	251.5 ± 55.2 (1)
0.2	15	1:0.3	0.321	48.9 ± 12.9 (92.8)	5225 ± 793.5 (5.8)
0.2	15	1:0.5	0.302	42.6 ± 21.7 (98)	3125 ± 85.5 (2)

**Table 3:** Follow-up of the colloidal stability of Ag NPs samples synthesized at different conditions after three months of synthesis: hydrodynamic diameter (DLS data).

Sample Silver (mol/L)	Time in Treatment (min)	AgNO_3_:Mesquite Gum Ratio	PdI	Size (d.nm) ± SE (% Volumen)	
				1st Peak	2nd Peak
0.01	15	1:1	0.380	35.6 ± 17.3 (77.3)	5462 ± 634.8 (18.7)
0.05	15	1:1	0.141	28.4 ± 9.3(100)	-
0.1	15	1:1	0.179	31.4 ± 6.5 (100)	-
0.2	15	1:1	0.237	30.3 ± 5.8 (100)	-
0.5	15	1:1	0.195	32.1 ± 10.5 (100)	-
0.01	30	1:1	0.237	51.8 ± 11.7 (100)	-
0.01	60	1:1	0.149	10.1 ± 2.1 (94.7)	53.3 ± 5.3 (5.3)
0.05	30	1:1	0.204	31.2 ± 6.3 (100)	-
0.05	60	1:1	0.378	26.7 ± 12.4 (94.4)	5456 ± 636.8 (5.6)
0.2	15	1:0.3	0.402	88.9 ± 5.6 (92.8)	7225 ± 954.7 (8.2)
0.2	15	1:0.5	0.398	62.9 ± 18.7 (95)	3125 ± 85.5 (5)

**Table 4:** Silver ion concentration by ion selective electrode measure and assessment of % Ag**^0^**.

Sample Name, Reaction Time, Diluted	Total Ag (M)	Electrode Potential (mV)	Ag^+^ (M)	Ag^0^ (M)	Concentration of Ag^0^ (%)
0.01 30 min 1:10	0.00093819	201.5	7.67183 × 10^−6^	0.000930516	99.1822713
0.01 30 min 1:100	0.00009382	197	2.78488 × 10^−7^	9.35403 × 10^−5^	99.70316447
0.01 60 min 1:10	0.00093819	250	6.43172 × 10^−6^	0.000931756	99.31445266
0.01 60 min 1:100	0.00009382	168.6	5.17802 × 10^−8^	9.3767 × 10^−5^	99.94480825
0.05 30 min 1:10	0.00416826	230.3	2.00221 × 10^−6^	0.004166256	99.95196538
0.05 30 min 1:100	0.00041683	198.3	3.00781 × 10^−7^	0.000416525	99.92784012
0.05 60 min 1:10	0.00416826	243.1	4.27378 × 10^−6^	0.004163984	99.89746842
0.05 60 min 1:100	0.00041683	201.7	3.67893 × 10^−7^	0.000416458	99.91173937
0.1 1:10	0.00797664	180.3	1.03554 × 10^−7^	0.007976537	99.99870178
0.1 1:100	0.00079766	190.6	1.90614 × 10^−7^	0.000797473	99.97610351
0.2 1:10	0.01519171	155	2.31355 × 10^−8^	0.015191687	99.99984771
0.2 1:100	0.00151917	210.2	6.08695 × 10^−7^	0.001518562	99.95993242
0.5 1:10	0.03312199	212.8	7.10051 × 10^−7^	0.03312128	99.99785626
0.5 1:100	0.0033122	221	1.15412 × 10^−6^	0.003311045	99.96515559

**Table 5:** Comparison of the estimated production capacity for several methods of Ag nanoparticle synthesis reported in the literature and the method reported here.

Authors	Method	Estimated Production Capacity (g Ag/100 g) *	Reaction Time	Particle Size	Observations	Reference
Moreno & Sánchez	Aqueous, Tollens with mesquite gum	~3.0 (with 0.5 M AgNO_3_)	~15–30 min	~30 nm	High colloidal stability in water (tested for 3 months)	This work
He et al.	Aqueous, Tollens, glucose, Al(NO_3_)_3_	~0.43	60 min	Nanosheets 27 nm thickness	Dimension of nanosheets, hundreds of nm	[25]
Kora et al.	Aqueous, kondagogu gum, Autoclave 121 °C	~0.01	~20–60 min	Various, 3–20 nm	Need high temperature and pressure	[63]
Dong et al.	Aqueous, Acacia (Arabic) gum, 60–80 °C	~0.06	~3 h	2–20 nm	High colloidal stability in water (tested for 1 month)	[64]
Kora et al.	Aqueous, ghatti gum, Autoclave 121 °C	~0.01	~20–60 min	Variou, 5–30 nm	Need high temperature and pressure	[55]
Sosa et al.	W/O microemulsion, 70 °C, NaBH_4_	~1.5	~2.5 h	9 nm	Toluene, AOT, SDS, 7 washings with water/acetone	[65]
Melendrez et al.	Ethyleneglycol, PVP, microwave	~1.0	~10 min	Nanowires 70–110 nm diameter	Need microwave treatment, solvent (ethyleneglycol)	[66]
He et al.	Aqueous, 12–2-12 gemini surfactant, NaBH_4_	~0.54	~10 h	11 nm	12–2-12 surfactant not available commercially	[67]
Li et al.	Tween^®^ 80, 100 °C	~3.2	1–3 days	Various, 20–40 nm	High surfactant concentration (about 95%); high temperature and large reaction times	[68]
Toisawa et al.	Ethanol, toluene, dodecylamine Ag_2_O powder, ultrasound	2–9	3–10 h	Various, 10–30 nm	Several hours of ultrasound needed; toluene used	[69]

* Estimated by the authors of the present work, taking into account the experimental data provided in the corresponding references.

## Supplementary Materials

The following are available online at www.mdpi.com/1996-1944/9/10/817/s1, Figure S1: Rheological behavior of various types of purified mesquite gums and Arabic gum, Figure S2: Thermogravimetric graph of various types of purified mesquite gums and Arabic gum, Figure S3: FT-IR spectra graph of various types of purified mesquite gums and Arabic gum, Table S1: Data table with the Newtonian model rheological parameters of the different samples of lyophilized mesquite gum and Arabic, Table S2: Data table with the TGA parameters of the different samples of lyophilized mesquite gum and Arabic.
