# Supplementary material for: Mesquite Gum as a Novel Reducing and Stabilizing Agent for Modified Tollens Synthesis of Highly Concentrated Ag Nanoparticles
Source: Materials (Basel). 2016 Oct 4;9(10):817. doi: 10.3390/ma9100817 (PMC5456612; doi:10.3390/ma9100817)
Supplement: Supplementary file 1 [file materials-09-00817-s001.docx]

Supplementary Materials: Mesquite Gum as a Novel Reducing and Stabilizing Agent for Modified Tollens Synthesis of Highly Concentrated Ag Nanoparticles

Maira B. Moreno-Trejo and Margarita Sánchez-Domínguez


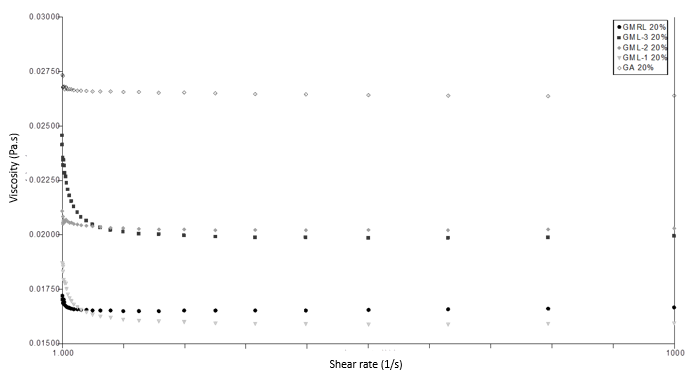


**Figure S1.** Rheology of various types of purified mesquite gums and Arabic gum.
Note: GML-1: Lyophilized mesquite gum with an initial concentration of 20% (sponge texture); GML-2: mesquite gum with dense texture (initial concentration 20%); GML-3: mesquite gum with methodology (diluted) at a concentration of 20% (initial concentration 40%), GMRL: mesquite gum with an initial concentration of 50% but subject to second process of freeze-drying; GA: commercial Arabic gum. The observed behavior is identical for the different samples of mesquite gum and Arabic gum, with a small difference in viscosity, which was expected due to the difference in constitution polysaccharide groups.

**Table S1.** Data table with the Newtonian model rheological parameters of the different samples of lyophilized mesquite gum and Arabic.

| **Sample** | **n (Pa*s)** | **Standard Deviation** |
| --- | --- | --- |
| GML-1 | 0.02 | 1 |
| GML-2 | 0.02 | 0.6 |
| GML-3 | 0.02 | 1.1 |
| GMRL | 0.017 | 0.92 |
| GA | 0.026 | 0.5 |

Note: Corresponds to Figure S1.

**Figure S2.** Thermogravimetric graph of various types of purified mesquite gums and Arabic gum.

**Table S2.** Data table with the TGA parameters of the different samples of lyophilized mesquite gum and Arabic.

| **Sample** | **Compound to Which Is Attributed** | **Temperature Range (°C)** |
| --- | --- | --- |
| GMH | Humidity | 60–100 |
|  | Polysaccharides | 200–400 |
| GML-1 | Humidity | 60–100 |
|  | Polysaccharides | 200–400 |
| GML-2 | Humidity | 60–100 |
|  | Polysaccharides | 200–400 |
| GML-3 | Humidity | 60–100 |
|  | Polysaccharides | 200–400 |
| GA | Humidity | 60–100 |
|  | Polysaccharides | 200–400 |

Note: Corresponds to Figure S2.


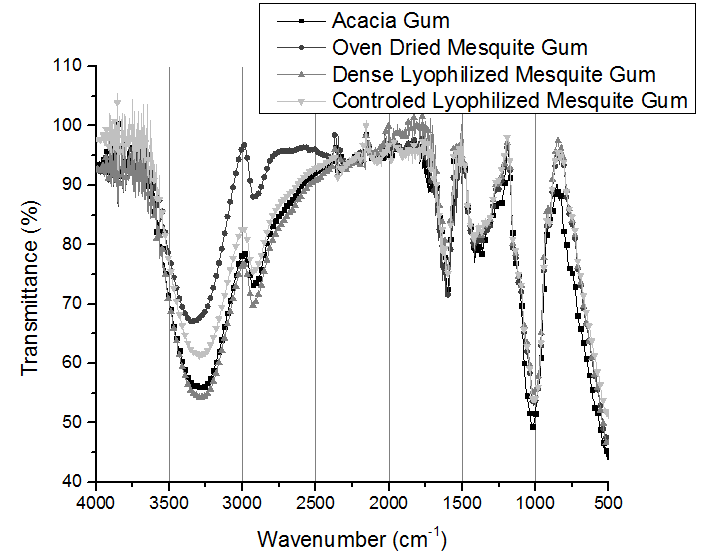


**Figure S3.** FT-IR spectra graph of various types of purified mesquite gums and Arabic gum. Note: Generally, the adsorptions of OH and CH groups are at 3375 cm^−1^ and 2932 cm^−1^, and a band centered between 1650 cm^−1^ and 1600 cm^−1^ that can be attributed to the primary amides. There is a small band around 1500 cm^−1^, which is assigned to the substitute or secondary amides. The bands of the primary and secondary amides are a characteristic of the presence of peptide bonds and confirm the presence of the protein in the sample. There is also a band at 1400 cm^−1^ that can be attributed to a carboxylic group. The bands that are around 1000 cm^−1^ and 900 cm^−1^ can be attributed to the pyranose glycosidic acetal groups, according to the literature [1,2].
